# CNNH_PSS: protein 8-class secondary structure prediction by convolutional neural network with highway

**DOI:** 10.1186/s12859-018-2067-8

**Published:** 2018-05-08

**Authors:** Jiyun Zhou, Hongpeng Wang, Zhishan Zhao, Ruifeng Xu, Qin Lu

**Affiliations:** 1 School Computer Science and Technology, Harbin Institute of Technology Shenzhen Graduate School, HIT Campus Shenzhen University Town, Xili, Shenzhen, Guangdong 518055 China; 2 Department of Computing, the Hong Kong Polytechnic University, Hung Hom, Hong Kong

**Keywords:** Protein secondary structure, Convolutional neural network, Highway, Local context, Long-range interdependency

## Abstract

**Background:**

Protein secondary structure is the three dimensional form of local segments of proteins and its prediction is an important problem in protein tertiary structure prediction. Developing computational approaches for protein secondary structure prediction is becoming increasingly urgent.

**Results:**

We present a novel deep learning based model, referred to as CNNH_PSS, by using multi-scale CNN with highway. In CNNH_PSS, any two neighbor convolutional layers have a highway to deliver information from current layer to the output of the next one to keep local contexts. As lower layers extract local context while higher layers extract long-range interdependencies, the highways between neighbor layers allow CNNH_PSS to have ability to extract both local contexts and long-range interdependencies. We evaluate CNNH_PSS on two commonly used datasets: CB6133 and CB513. CNNH_PSS outperforms the multi-scale CNN without highway by at least 0.010 Q8 accuracy and also performs better than CNF, DeepCNF and SSpro8, which cannot extract long-range interdependencies, by at least 0.020 Q8 accuracy, demonstrating that both local contexts and long-range interdependencies are indeed useful for prediction. Furthermore, CNNH_PSS also performs better than GSM and DCRNN which need extra complex model to extract long-range interdependencies. It demonstrates that CNNH_PSS not only cost less computer resource, but also achieves better predicting performance.

**Conclusion:**

CNNH_PSS have ability to extracts both local contexts and long-range interdependencies by combing multi-scale CNN and highway network. The evaluations on common datasets and comparisons with state-of-the-art methods indicate that CNNH_PSS is an useful and efficient tool for protein secondary structure prediction.

## Background

The concept of secondary structure was first introduced by Linderstrøm-Lang at Stanford in 1952 [1, 2] to represent the three dimensional form of local segments of proteins. Protein secondary structure is defined by the pattern of hydrogen bonds between the amine hydrogen and carbonyl oxygen. There are two ways used for the classification of protein secondary structures: three-category classification(Q3) and eight-category classification(Q8). Q3 classifies target amino acid residues into helix(H), strand(E) and coil(C) while Q8 classifies target amino acid residues into (1) 3-turn helix(G), (2) 4-turn helix(H), (3) 5-turn helix(I), (4) hydrogen bonded turn(T), (5) extended strand in parallel and/or anti-parallel β-sheet conformation(E), (6) residue in isolated β-bridge (B), (7) bend(S) and (8) coil(C) [3–5]. Most protein secondary structure prediction studies have been focused Q3 prediction. Q8 prediction is more challenging and can reveal more structural details [6, 7], so we focus the Q8 prediction in this study.

Protein secondary structure prediction is secondary structure inference of protein fragments based on their amino acid sequence. In bioinformatics and theoretical chemistry, protein secondary structure prediction is very important for medicine and biotechnology, for example drug design [8] and the design of novel enzymes. Since secondary structure can be used to find distant relationship for proteins with unalignable primary structures, incorporating both secondary structure information and simple sequence information can improve the accuracy of their alignment [9]. Finally, protein secondary structure prediction also plays an important role in protein tertiary structure prediction. Protein secondary structure can determine the structure types of protein local fragments, so the freedom degree of protein local fragments in the tertiary structure can be reduced. Therefore accurate secondary structure prediction is potential for improving the accuracy of protein tertiary structure prediction [4, 7, 10].

Three experimental methods were proposed to determine secondary structures for proteins: far-ultraviolet circular dichroism, infrared spectroscopy and NMR spectrum. Far-ultraviolet circular dichroism predict pronounced double minimum at 208 and 222 nm as α-helical structure and single minimum at 204 nm or 217 nm as random-coil or β-sheet structure, respectively [11]. Infrared spectroscopy uses the differences in the bond oscillations of amide groups for prediction [12] while NMR spectrum predict protein secondary structure by using the estimated chemical shifts [12]. As experimental methods are costly and the proteins with known sequence continue to outnumber the experimentally determined secondary structures, developing computational approaches for protein secondary structure prediction becomes increasingly urgent. Existing computational approaches for protein secondary structure prediction can be divided into 3 categories. The first category is statistical model based methods, which can date back to 1970s. Early, this category uses statistical models to analyze the probability of secondary structure elements for individual amino acid residue [13]. Next, the statistical models were applied for the prediction of segments of 9–21 amino acids. For example, the GOR method [14] used amino acid segment to predict the structure of its central residue. However, the performances (< 60% Q3 accuracy) of this category of methods are far from practical application due to inadequate features.

Due to the lacking of inadequate features for the statistical model based methods, evolutionary information based methods have been proposed. These methods usually used the evolutionary information of proteins from a same structural family [15] extracted by multiple-sequence alignment or position-specific scoring matrices (PSSM) [16] from PSI-BLAST for prediction. An earlier evolutionary information based method was developed based on a two-layered feed-forward neural network, for which the evolutionary information in the form of multiple sequence alignment is used as input instead of single sequences [15]. As SVM [17] is significantly better than neural network in a wide range of pattern recognition problems [18–21], Hua and Sun first proposed a SVM classifier for protein secondary structure prediction [22]. The input for SVM is evolutionary information in the form of multiple sequence alignment. Unbalanced data is a challenging problem in protein secondary structure prediction and existing methods lack the ability to handle it [23, 24]. So Kim and Park proposed a new protein secondary structure prediction method, SVMpsi, by an improved SVM, which reduces the influence of imbalanced data by using different penalty parameters in the improved SVM [23]. By using different penalty parameters, SVMpsi resolved the situation where the recall value of the smaller class is too small. Another SVM based method is PMSVM which was proposed by using dual-layer support vector machine (SVM) and evolutionary information in form of PSSMs [25].

Protein sequence usually contains two types of sequence information: local context and long-range interdependencies [4, 26, 27]. Local contexts denote the correlations between residues with distance less than or equal to a predefined threshold while long-range correlation are the correlations between residues with distance more the threshold. Inspired by the success of convolutional neural networks (CNN) [28] for local context extraction in natural language processing tasks [29, 30], multi-scale CNN has been used to capture local contexts for protein secondary structure prediction [31]. For example, Wang et al. proposed conditional neural fields (CNF) [7] to extract local contexts for prediction by integrating a windows-based neural network with conditional random field (CRF). In addition to local contexts, long-range interdependencies also are important for protein secondary structure prediction(BRNN) [6, 32–34]. In order to extract both local contexts and long-range interdependencies for prediction, Zhou and Troyanskaya proposed GSN [4] by using convolutional architecture and supervised generative stochastic network, which is a recently proposed deep learning technique [26]. In addition to GSN, a novel deep convolutional and recurrent neural network (DCRNN) also has been proposed by Li and Yu [27] for protein secondary structure prediction by extracting both local contexts and long-range interdependencies.

In summary, the statistical model based methods and evolutionary information based methods cannot extract local contexts and long-range interdependencies for prediction. For the deep learning based methods, some methods cannot extract both local contexts and long-range interdependencies for prediction. Although several methods can extract both local contexts and long-range interdependencies, such as GSN and DCRNN, they need extra complex models to extract long-range interdependencies, which are complex and time-consuming. In this paper, we propose a novel method, referred to as CNNH_PSS, by combining multi-scale CNN with highway network, which has ability to extract both local contexts and long-range interdependencies without needing extra models. CNNH_PSS consists of two parts: multi-scale CNN and fully connected and softmax layer. In the multi-scale CNN, any two neighbor convolutional layer contains a highway to deliver information from current layer to the output of the next one to keep local contexts. As the convolutional kernels in higher layer can extract long-range interdependencies by using the local contexts extracted by lower layers, thus with the layer number increasing, CNNH_PSS can extract long-range interdependencies covering more remote residues while keeping local contexts extracted by lower layers by using highway. So CNNH_PSS can extract both local contexts and long-range interdependencies covering very remote residues for prediction. The source code of our proposed method CNNH_PSS is provided for free access to the biological research community at http://hlt.hitsz.edu.cn/CNNH_PSS/ and http://119.23.18.63:8080/CNNH_PSS/.

## Methods

As shown by many recently published works [35–37], a complete prediction model in bioinformatics should contain the following four components: validation benchmark dataset(s), an effective feature extraction procedure, an efficient predicting algorithm, a set of fair evaluation criteria. In the following text, we will describe the four components of our proposed CNNH_PSS in details.

### Datasets

Two publicly available datasets: CB6133 and CB513 were used to evaluate the performance of our proposed method CNNH_PSS and compare with state-of-the-art methods.

#### CB6133

CB6133 was produced by PISCES CullPDB [38] and is a larger non-homologous protein dataset with known secondary structure for every protein. It contains 6128 proteins, in which 5600 proteins are training samples, 256 proteins are validation samples and 272 proteins are testing samples. This dataset is publicly available from literature [4].

#### CB513

CB513 is a public testing dataset and can be freely obtained from [4, 39]. For the testing on CB513, CB6133 is used as the training dataset. As there exists redundancy between CB513 and CB6133, CB6133 is filtered by removing sequences having over 25% sequence similarity with sequences in CB513. After filtering, 5534 proteins left in CB6133 are used as training samples. Since GSN [4] and DCRNN [27] as well as other state-of-the-art methods [31, 40] performed a validation on CB513 to get their best performance, we also perform a validation on CB513 to get the best performance of our method CNNH_PSS to make fair comparisons with them.

### Feature representation

Given a protein with *L* amino acid residues as *X* = *x*_1_, *x*_2_, *x*_3_, ⋯, *x*_*L*_, where *x*_*i*_(∈ℝ^*m*^) is the *m*-dimensional feature vector of the *i*^th^ residue, the secondary structure prediction for this protein is formulated as determining *S* = *s*_1_, *s*_2_, *s*_3_, ⋯, *s*_*L*_ for *X* where *s*_*i*_ is a Q8 secondary structure label. In this study,*x*_*i*_ is encoded by both sequence features and evolutionary information. Sequence features are used to specify the identity of the target residue. Two methods are used to encode sequence features: one hot and residue embedding. One hot encodes sequence features of each residue by a 21-dimension one-hot vector, in which only one element equals to 1 and the remaining elements are set to 0, where 21 denotes the 20 standard types of residues and one extra residue type which represents all non-standard residue types. However, one-hot vector is a sparse representation and unsuitable for measuring relation between different residues. In order to get dense representation of sequence features, an embedding technique in natural language processing is used to transform 21-dimensional one-hot vector to a 21-dimensional denser representation [41]. The embedding technique maps words or phrases from the vocabulary to vectors of real numbers. Specifically, it maps words from a space with one dimension per word to a continuous vector space with much lower dimension. So the embedding technique provides a real value for every dimension. As the dimension of amino acid representation is already low, we only calculate a real value for every dimension by embedding technique and don’t decrease the dimension. The residue embedding in this paper is implemented by a feedforward neural network layer before multi-scale CNN in CNNH_PSS [42].

Evolutionary information such as position-specific scoring matrix (PSSM) is considered as informative features for predicting secondary structure by previous research [16]. PSSM is a common representation for evolutionary information and has been used in many bioinformatics studies including protein functionality annotation and protein structure prediction [43–47]. In this study, PSSM is calculated by PSI-BLAST [48] against the UniRef90 database with E-value threshold 0.001 and 3 iterations. UniRef90 database contains the known protein sequences with sequence identity less than 90 from almost all known species. So the PSSM calculated from UniRef90 database contains the common sequence information among the known protein sequences of different species. Overtime, scientists have reached a consensus that a protein’s structure primarily depends on its amino acid sequence and concluded that the local and long-range interaction are a cause of protein second and tertiary structure. Based on this hypothesis, we can deduce that proteins with similar amino acid sequence tend to have similar secondary structure sequence. Therefore, the common sequence information contained by PSSM can contribute to the secondary structure prediction. For a protein with length *L*, PSSM is usually represented as a matrix with *L* × 21 dimensions where 21 denotes the 20 standard types of residues and one extra residue type which represents all non-standard residue types. Before PSSMs are used inputs for CNNH_PSS, they need to be transformed to 0–1 range by the sigmoid function. By concatenating sequence features and evolutional information, each residue in protein sequences can be encoded by a feature vector with dimension of 42.

### Multi-scale CNN with highway between neighbor layers

**Fig. 1:**
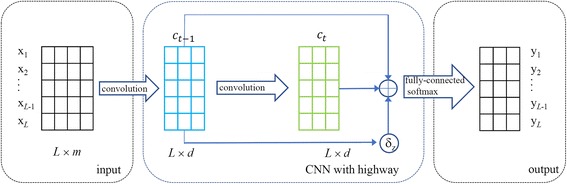
The frame of CNNH_PSS

In CNN model, a kernel can examine a local patch in input sequence and extract interdependence among the residues contained in the local patch. With stacking of convolutional layers, the kernels for deep layers have ability to cover correlations among more spread-out residues in the input sequence. So, CNN model with more number of convolutional layers have the ability to extracted long-range interdependencies between residues with more large distance. However, with the number of layers increasing, CNN model will lose the local contexts extracted by lower layers. In this paper, we propose a novel method, referred to as CNNH_PSS, to resolve this problem. CNNH_PSS contains a highway between any two neighbor convolutional layers in multi-scale CNN. As the number of convolutional layers increases, CNNH_PSS can not only extract long-range interdependencies by higher layers, but also obtain the local contexts extracted by lower layers through highway. The frame of CNNH_PSS is shown in Fig. 1. Figure 1 shows that CNNH_PSS contains three parts: input section, multi-scale CNN with highway and output section. In the input section, *x*_*i*_ ∈ R^*m*^ denotes the feature vector of the *i*^th^ residue in protein, which is the concatenation of sequence features and evolutional information. Thus a protein of length *L* is encoded as a *L* × *m* matrix *x*_1 : *L*_ = [*x*_1_, *x*_2_, ⋯, *x*_*L*_]^T^, where *L* and *m* denote the length of protein and the number features used to encode residues, respectively. In this study, *m* equals to 42. In order to keep the output of convolutional layer have the same height with the input, we need to pad ⌊*h*/2⌋ and ⌊(*h* − 1)/2⌋ *m*-dimensional zero vectors to the head and the tail of the input *x*_1 : *L*_, respectively, where *h* is the length of convolutional kernels in the convolutional layer. The second section contains two parts: multi-scale CNN and highway, where the multi-scale CNN contains *n* convolutional layers. In the (*t* − 1)^th^ layer, the convolution operation of the *k*^th^ kernel ${w}_k^{t-1}\in {\mathrm{R}}^{h\times m}$ executed on protein fragment *x*_*i* : *i* + *h* − 1_ is expressed as

1 $$ {c}_{k,i}^{t-1}={f}^{t-1}\left({w}_k^{t-1}\cdot {x}_{i:i+h-1}+{b}_k^{t-1}\right) $$

where *h* is the length of convolution kernel,${b}_k^{t-1}$ is the bias of the *k*^th^ kernel, *f* is activation function and *x*_*i* : *i* + *h* − 1_ denotes the protein fragment *x*_*i*_, *x*_*i* + 1_, *x*_*i* + 2_, ⋯, *x*_*i* + *h* − 1_. Through executing convolution operation of the *k*^th^ kernel on all fragments with length *h* of the padded input, we get a novel feature vector

2 $$ {c}_k^{t-1}={\left[{c}_{k,1}^{t-1},{c}_{k,2}^{t-1},{c}_{k,3}^{t-1},\cdots, {c}_{k,L}^{t-1}\right]}^{\mathrm{T}} $$

Suppose we have *d* kernels in the convolutional layer, thus we can get *d* novel features vectors. By concatenating the *d* novel feature vectors, we can get a novel feature matrix with dimension *L* × *d*

3 $$ {c}^{t-1}=\left[{c}_1^{t-1},{c}_2^{t-1},{c}_3^{t-1},\cdots, {c}_L^{t-1}\right] $$

This novel feature matrix is used as the input of the next convolutional layer. If there are *n* convolutional layers and *θ*_*t*_ is used to denote the kernels and the bias of the *t*^*th*^ convolutional layer, then the output of the *n*^*th*^ convolutional layer is

4 $$ {c}^n={f}_{\theta_n}^n\left({f}_{\theta_{n-1}}^{n-1}\left(\cdots {f}_{\theta_1}^1\left({x}_{1:L}\right)\right)\right) $$

Finally, the output of the *n*^*th*^ convolutional layer is used as the input of the fully connected softmax layer for prediction

5 $$ {y}_i= argmax\left(w\cdot {c_i}^n+b\right) $$

where *w* and *b* is the weight and bias of the fully connected softmax layer, respectively. *c*_*i*_^*n*^ is the feature vector of the *i*^*th*^ outputted by the *n*^*th*^ convolutional layer and *y*_*i*_ is its predicted secondary structure.

CNN has achieved huge progress in many tasks of image processing filed, one common sense is that the successes of CNN are attributed to the multiple convolutional layers in CNN, because CNN with more number of layers can extract correlations covering more residues. However, with the increasing of number of layers in CNN, the information communication between layers will become more difficult and the gradient will disappear [49]. Furthermore, the local contexts extracted by lower layers also will lose. Srivastava et al. [49] have proposed highway network to resolve these problems. So CNNH_PSS incorporates highway network and multi-scale CNN to extract both local contexts and long-range interdependencies for secondary structure prediction.

In CNNH_PSS, each convolutional layer except the last layer has three accesses to the next layer (shown in Fig. 1). Two accesses are used to deliver information from the current layer to the output and convolution kernels of the next layer, respectively. The other one is a weight used to determine the share of information in for information from highway. So the output *c*^*t*^ of the *t*^*th*^ convolutional layer is the weighted sum of the information delivered by highway from last layer and that outputted by the convolution kernels of current layer

6 $$ {z}_t=\delta \left({w}^z{c}^{t-1}\right) $$

7 $$ {c}^t=\left(1-{z}_t\right)\times {f}_{\theta_t}^t\left({c}^{t-1}\right)+{z}_t\times {c}^{t-1} $$

where *δ*(⋅) is *sigmoid* function,*z*_*t*_ is the weight of the highway and ${f}_{\theta_t}^t\left(\cdot \right)$ is the convolution operation of current convolutional layer. So the output of the*t*^*th*^convolutional layer contains two portion: information from the (*t* − 1)^*th*^convolutional layer delivered by highway and that outputted by the convolution kernels of current layer.

## Results

The purpose of the evaluation is to examine the effectiveness of our proposed CNNH_PSS over other methods. Four sets of evaluations are conducted here. The first experiment evaluates the performance of multi-scale CNN on CB6133 and CB513. The second experiment evaluates our proposed method CNNH_PSS on CB6133 and CB513. The third experiment compares CNNH_PSS with state-of-the-art methods. Finally, based on CB6133, we analyze the local contexts and long-range interdependencies learned by CNNH_PSS. As we mainly focus the Q8 prediction of protein secondary structure in this study, the performances of prediction methods are measured by Q8 accuracy [4, 27]. The Q8 accuracy is the percentage of the amino acid residues for which the predicted secondary structure labels are correct. The source code of our proposed method CNNH_PSS is provide for free access at http://hlt.hitsz.edu.cn/CNNH_PSS/.

### The performance of multi-scale CNN model

**Fig. 2:**
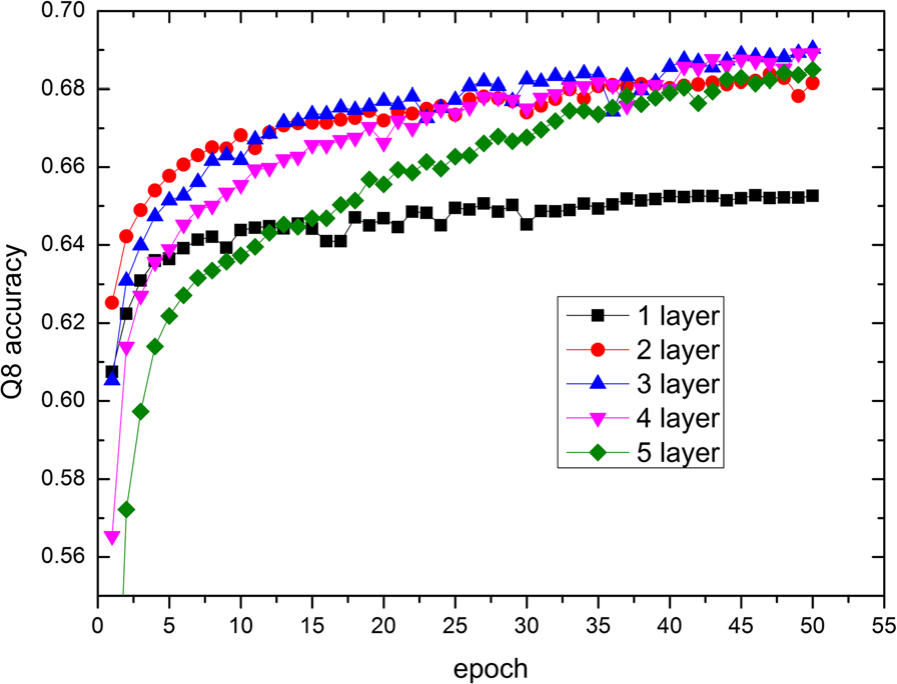
The performance of multi-scale CNN with different number of convolutional layers

In this section, multi-scale CNN is used to predict secondary structure for proteins. The hyper-parameters of the multi-scale CNN for protein secondary structure prediction in this study are listed in Table 1. Note that three kernel lengths are used in the multi-scale CNN model and 80 kernels are used for each kernel length. To conveniently encode and process protein sequences, the length of all protein sequences are normalized to 700. When sequences are shorter than 700, they will be padded with zero vectors. And when sequences are longer than 700, they will be truncated. In order to get best performance, we need to determine how many convolutional layers the multi-scale CNN should contains. We conduct experiments to evaluate the performances of the multi-scale CNNs with different number of convolutional layers on CB513. The performances are shown in Fig. 2, where the *x*-axis is the number of epochs used to train multi-scale CNN and the *y*-axis is Q8 accuracy. Fig. 2 shows the performances for models with number of convolutional layers from 1 to 5. From this figure, we see that the model with 3 convolutional layers gets the best accuracy. When the number of convolutional layers is increased to 4 or 5, the accuracy is decreased obviously. The main reason for this phenomenon may be the loss of extracted local contexts with the increasing of the number of convolutional layers in CNN. With the increasing of the number of convolutional layers in CNN, the correlations extracted by higher can cover more residues so that they contains interdependencies between more remote residues. When the number of convolutional layers is increased to 3, the CNN may achieve both local contexts and long-range interdependencies, which is validated by that the CNN with 3 convolutional layers gets the best accuracy in our problem. However, when the number of convolutional layers is more than 3, most local contexts extracted by lower layers are lost in the transport processes of information cross layers, causing the relationships outputted by the last layer in CNN contains less and less local contexts. So the predicting accuracy starts to decrease when the number of convolutional layers is more than 3.

**Table 1:** Hyper-parameters of multi-scale CNN

Layer	Hyper-parameter	Value
Multi-scale CNN	Kernel length	[7, 9, 11]
	Number of kernels	80 for each kernel length
	Batch size	50
	Learning rate	2e-3
	Regularizer	5e-5
	Decay rate	0.05
	Activation function	ReLU

The performances of multi-scale CNN with 3 convolutional layers on CB6133 and CB513 are shown in Table 2, where two sequence features encoding methods for residues are evaluated: one hot and residue embedding. Table 2 shows that residue embedding outperforms one hot on both CB6133 and CB513 by at 0.004 Q8 accuracy, indicating that residue embedding is a better encoding method for sequence features of residues. In the following text, we will use residue embedding method to encode sequence features in the multi-scale CNN model and our proposed method CNNH_PSS.

**Table 2:** The Q8 accuracy of Multi-scale CNN with 3 convolutional layers

datasets	CB6133	CB513
Multi-scale CNN(one hot)	0.721	0.689
Multi-scale CNN(embedding)	*0.729*	*0.693*

The data in italic denote the best performance

### The performance of CNNH_PSS

Local contexts are the relationships among residues at close range while long-range interdependencies are the relationships among remote residues. As there is no strict bounds between local contexts and long-range interdependencies, we specify the information extracted by the first convolutional layers as local contexts and that extracted by all other layers as long-range interdependencies in this study. In CNNH_PSS, any two neighbor convolutional layers have a highway to deliver information from current convolutional layer to the output of the next one, so it can make sure that the output of each layer contains a portion of the local contexts. Furthermore, the convolution kernels of each layer except the first one can extract long-range interdependencies by using the information from previous layer. With the increasing of the number of convolutional layers, CNNH_PSS can extract long-range interdependencies between more remote residues. Therefore, the output the last convolutional layer in CNNH_PSS contains two portion of information: local contexts extracted by the first layer and long-range interdependencies extracted by all other layers.

**Fig. 3:**
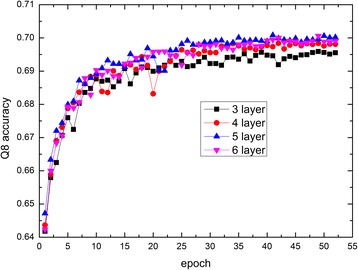
The performance of CNNH_PSS with different number of convolutional layers

Similarly, we evaluate the performances of our proposed method CNNH_PSSs with different number of convolutional layers on CB513. The performances are shown in Fig. 3. Figure 3 shows that CNNH_PSS achieves the best performance when the number of convolutional layers is 5. When number of convolutional layers is more than 5, the performance of our method starts to decrease. So CNNH_PSS with 5 convolutional layers is used in the following text. Comparing the multi-scale CNN model with 3 convolutional layers described in above section, our proposed method CNNH_PSS not only contains a highway between any two neigbouring layers, but also have more number of convolutional layers. It makes sure that CNNH_PSS with 5 layers can not only extract local contexts, but also capture long-range interdependencies between more remote residues than the multi-scale CNN model with 3 layers. The performances of CNNH_PSS and the multi-scale CNN model on CB6133 and CB513 are shown in Table 3. Table 3 shows that our proposed method CNNH_PSS outperforms the multi-scale CNN model by 0.011 Q8 accuracy on CB6133 and 0.010 Q8 accuracy on CB513. The outperformance of CNNH_PSS over the multi-scale CNN model on both CB6133 and CB513 validates that the highway in CNN indeed are useful for protein secondary structure prediction.

**Table 3:** The Q8 accuracy of CNNH_PSS

Method	CB6133	CB513
Multi-scale CNN	0.729	0.693
CNNH_PSS	*0.740*	*0.703*

The data in italic denote the best performance

### Comparison with state-of-the-art methods

Protein secondary structure prediction is an important problem in bioinformatics and critical for analyzing protein function and applications like drug design. So many state-of-the-art methods have been proposed for the prediction. SSpro8 is a prediction method proposed by Pollastri et al. [6] by combining bidirectional recurrent neural networks (RNN) and PSI-BLAST-derived profiles. CNF is a Conditional Neural Fields based method which was proposed by Wang et al. [40], which can not only extract relationships between sequence features of residues and their secondary structures, but also capture local contexts [40]. Later, an extension version of CNF (DeepCNF) was proposed by Wang et al. [31] using deep learning extension of conditional neural fields, which is an integration of conditional neural fields and shallow neural networks. It can extract both complex sequence-structure relationship and interdependency between adjacent SS labels. These three methods can only extract local contexts for prediction. Furthermore, GSN is a prediction method proposed by Zhou and Troyanskaya [4] using supervised generative stochastic network and convolutional architectures. Supervised generative stochastic network is a recently proposed deep learning technique [26], which is well suitable for extracting local contexts and also can capture some long-range interdependencies. Finally, DCRNN is the best performing method up to now, which was recently proposed by Li and Yu [27] using multi-scale CNN and three staked bidirectional gate recurrent units (BGRUs) [50]. GSN and DCRNN can extract both local contexts and long-range interdependencies. We first compare our proposed method CNNH_PSS with the three state-of-the-art methods which only can extract local contexts on CB513. The performances of these three methods and our method on CB513 are listed in Table 4. Table 4 shows that CNNH_PSS outperforms the three methods by at least 0.020 Q8 accuracy. The outperformance of CNNH_PSS over the three state-of-the-art methods which only can extract local contexts indicates that the long-range interdependencies extracted by CNNH_PSS are indeed useful for protein secondary structure prediction.

**Table 4:** The Q8 accuracy of CNNH_PSS and state-of-the-art methods containing only local contexts

Method	CB513
SSpro8	0.511
CNF	0.633
DeepCNF	0.683
CNNH_PSS	*0.703*

The data in italic denote the best performance

Then, we compare our method CNNH_PSS to GSN and DCRNN by both CB6133 and CB513, which also can extract both local contexts and long-range interdependencies. The performances of these two methods and our method on CB6133 and CB513 are listed in Table 5. The table shows that CNNH_PSS performs better than GSN and DCRNN by at least 0.008 Q8 accuracy on CB6133 and 0.009 Q8 accuracy on CB513. GSN and DCRNN consist of CNN for local context extraction and extra models for long-range interdependencies extraction. As the extra models of the two methods are complex and time-consuming, these two methods need consume much computer resource. Trained on GTX TITANX GPU, CNNH_PSS tends to converge after only a half hour while DCRNN needs more than 24 h to converge [27]. So CNNH_PSS is almost 50 times faster than DCRNN. Although we do not known the exact running time for GSN, we know that GSN needs to be trained for 300 epochs [4] while CNNH_PSS tends to converge after training for less than 35 epochs shown by Fig. 3. It means that CNNH_PSS is almost 9 times faster then GSN. Therefore, the outperformance of our method over GSN and DCRNN further demonstrates that CNNH_PSS can not only cost less computer resource but also achieves better predicting performance.

**Table 5:** The Q8 accuracy of CNNH_PSS and state-of-the-art methods containing both local contexts and long-range interdependencies

Method	CB6133	CB513
GSN	0.721	0.664
DCRNN	0.732	0.694
CNNH_PSS	*0.740*	*0.703*

The data in italic denote the best performance

## Discussion

**Fig. 4:**
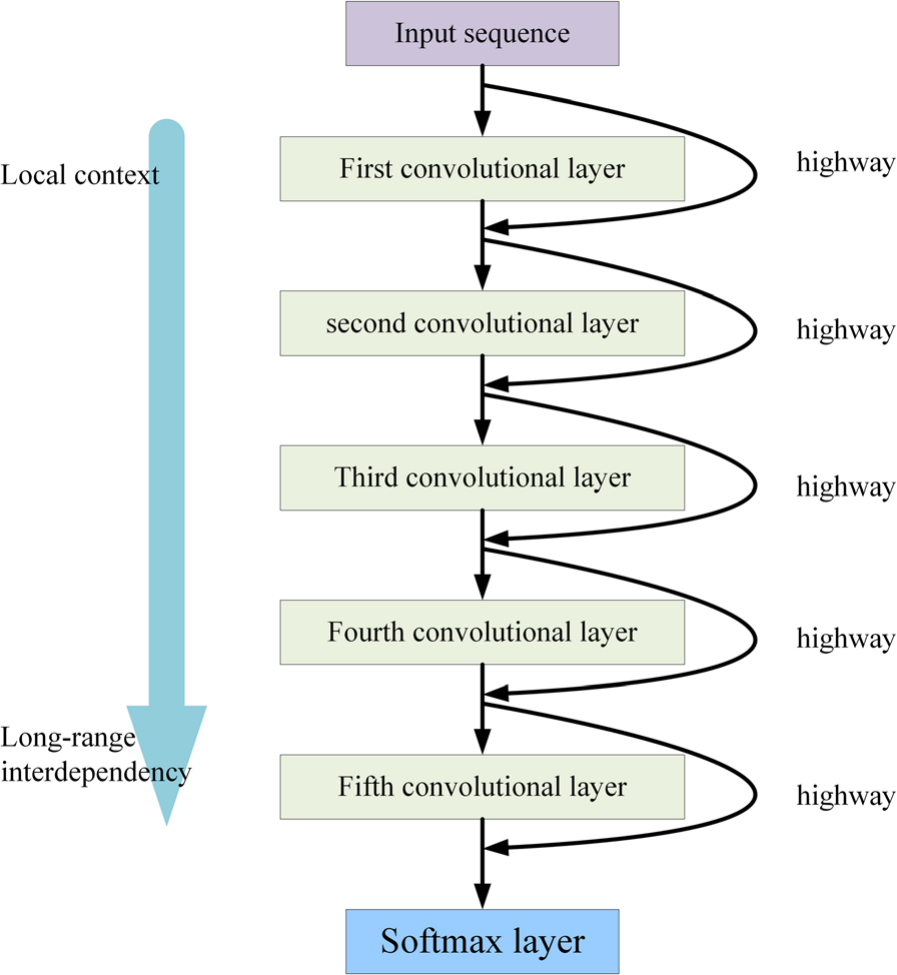
Extraction process for local contexts and long-range interdependencies

The advantage of our proposed method CNNH_PSS over state-of-the-art methods is that it can extract both local contexts and long-range interdependencies by using multi-scale CNN with highway. In CNNH_PSS, any two neighbor convolutional layers have a highway to deliver information from current convolutional layer to the output of the next one and each layer except the first one have convolution kernels to extract long-range interdependencies by using the information from previous layer. In this section, we use CNNH_PSS with 5 convolutional layers and the kernel length of 11 to introduce the process for local contexts and long-range interdependencies extraction, which is shown in Fig. 4. First, the target protein are inputted to the first layer and the convolution kernels in the first layer extract local contexts from the inputted protein. So the output of first layer contains local contexts among 11 residues. Then the information in the output of the first layer are delivered to the output of the second layer by two ways: highway between them and the convolution kernels in the second layer. Finally, the output of the second layer is the weighted sum of the information transmitted by the two way. As the convolution kernels in the secondary layer can extract relationships among 21 residues, the output of the second layer contains both local contexts among 11 residues and long-range interdependencies among 21 residues. And so on, the output of the fifth layer contains two parts. One part is the information from the output of the fourth layer by the highway between them, which contains local contexts among 11 and long-range interdependencies among 21, 31 and 41 residues. The other part is the information outputted by the convolutional kernels of current layer, which contains long-range interdependencies among 51 residues. Therefore CNNH_PSS can output local contexts among 11 and long-range interdependencies among 21, 31, 41 and 51 residues while the multi-scale CNN with the same number of convolutional layer as CNNH_PSS outputs only long-range interdependencies among 51 residues.

**Fig. 5:**
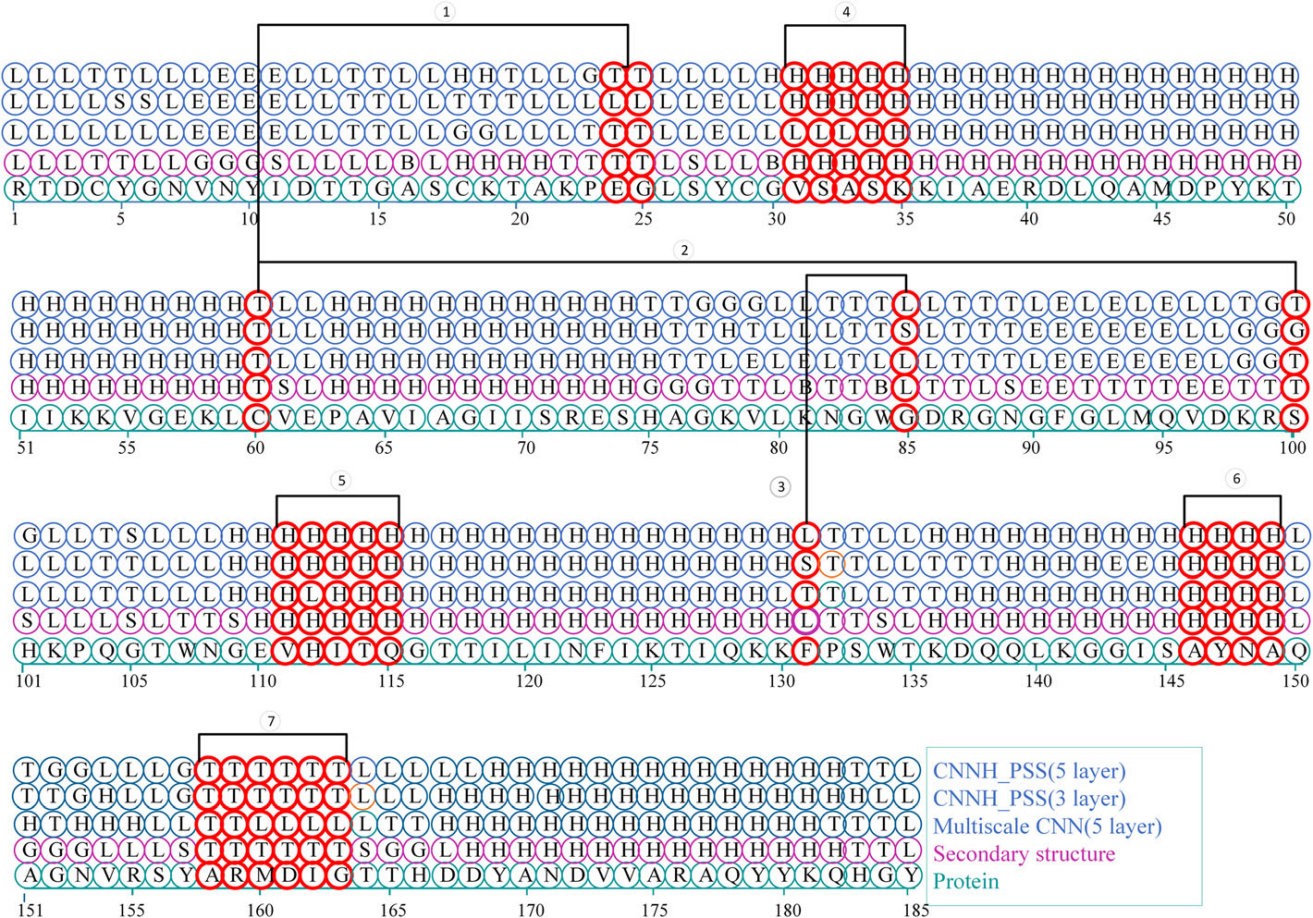
Prediction results of 154 L by CNNH_PSS

In order to demonstrate the importance of learned local contexts and long-range interdependencies in protein secondary structure prediction, we show the local contexts and the long-range interdependencies learned in a representative protein PDB 154 L [51], which obtained from the publicly available protein data bank [52]. The learned local contexts and long-range interdependencies by CNNH_PSS in protein PDB 154 L are shown in Fig. 5. In Fig. 5, the five rows correspond to the predicted results by CNNH_PSS with 5 layers, that by CNNH_PSS with 3 layers, that by the multi-scale CNN with 5 layers, real secondary structures and protein sequence, respectively. The reason for why CNNH_PSS with 5 layers, CNNH_PSS with 3 layers and the multi-scale CNN with 5 layers are selected for comparison is that CNNH_PSS with 5 layers can extracted local contexts and long-range interdependencies among up to 51 residues while CNNH_PSS with 3 layers cannot extracted long-range interdependencies among more than 41 residues and the multi-scale CNN with 5 layers cannot extract local contexts. Fig. 5 shows three instances for long-range interdependencies: (1) interdependencies among 24th, 25th and 60th amino acid; (2) that between 60th and 100th and (3) that between 85th and 131th amino acid. As the number of residues covered by these three learned interdependencies is more than 31 and less than 51 residues, both CNNH_PSS with 5 layers and the multi-scale CNN with 5 layers can extract them for correct prediction them while CNNH_PSS with 3 layers cannot capture them. So both CNNH_PSS with 5 layers and the multi-scale CNN with 5 layers make correct prediction for the 24th, 25th, 85th, 100th and 131th residues while CNNH_PSS with 3 layers cannot make correct predictions for them. It validates that CNNH_PSS with more layers indeed can extract long-range interdependencies between more remote residues.

Furthermore, Fig. 4 also shows 4 instances for learned local contexts: (1) contexts from 31th to 35th residues; (2) that from 111th to 115th residues; (3) that from 146th to 149th residues and (4) that from 158th to 163th residues. Both CNNH_PSS with 3 layers and that with 5 layers can learn these four contexts so that the secondary structures of all the residues in the learned contexts can be correctly predicted. However, the multi-scale CNN with 5 layers cannot learn these four contexts. So it cannot predict the secondary structures correctly for these residues. It validates that the highways in the CNNH_PSS indeed can be used to extract local contexts for prediction.

## Conclusion

Protein secondary structure prediction is an important problem in bioinformatics and critical for analyzing protein function and applications like drug design. Several experimental methods have been proposed to determined the secondary structures for proteins, such as far-ultraviolet circular dichroism, infrared spectroscopy and NMR spectrum. However, experimental methods usually are costly and time-consuming. And the proteins with known sequence continues to outnumber the experimentally determined secondary structures. So developing computational approaches that can accurately handle large amount of data becomes increasingly urgent. However, most of these proposed methods cannot extract either local contexts or long-range interdependencies. Although GSM and DCRNN can extract both of them, they are build by combing CNN architecture and extra complex models. Yet CNNH_PSS is developed by only multi-scale CNN with highway. So comparing to GSM and DCRNN, CNNH_PSS may cost less computer resource. We evaluate CNNH_PSS on two commonly used datasets: CB6133 and CB513. CNNH_PSS outperforms the multi-scale CNN without highway on both datasets, which demonstrates that the extracted local contexts through highways are indeed useful for protein secondary structure prediction. CNNH_PSS also outperforms CNF and DeepCNF as well as SSpro8 on CB513, which cannot extract long-range interdependencies. It indicates that long-range interdependencies extracted by CNNH_PSS are useful for protein secondary structure prediction. Furthermore, CNNH_PSS performs better than GSM and DCRNN, demonstrating that CNNH_PSS can not only cost less computer resource but also achieves better predicting performance than state-of-the-art methods. We also analyze the local contexts and long-range interdependencies learned by CNNH_PSS in protein PDB 154 L and show their roles in protein secondary structure prediction. X-ray diffraction crystallography and NMR can measure the structures of proteins, so these methods can be used to calculate the distances between any two residues in a protein sequence. By analyzing the second structures of long-range residues but with short distance in space and short-range residues, we can further demonstrate the importance of long-range interdependencies and local contexts for second structure prediction. Therefore, our future works will validate the conclusions achieved in this paper by using these experimental methods.

## Abbreviations

B: residue in isolated β-bridge
C: coil
CNN: convolutional neural network
CRF: conditional random field
E: extended strand in parallel and/or anti-parallel β-sheet conformation
G: 3-turn helix
GSN: generative stochastic network
H: 4-turn helix
I: 5-turn helix
NMR: nuclear magnetic resonance
PDB: Protein Data Bank
PSSM: Position Specific Score Matrix
Q3: three-category classification
Q8: eight-category classification
RNN: recurrent neural networks
S: bend
SVM: Support Vector Machine
T: hydrogen bonded turn
